# Marine Diterpenes: Molecular Modeling of Thrombin Inhibitors with Potential Biotechnological Application as an Antithrombotic

**DOI:** 10.3390/md15030079

**Published:** 2017-03-20

**Authors:** Rebeca Cristina Costa Pereira, André Luiz Lourenço, Luciana Terra, Paula Alvarez Abreu, Valéria Laneuville Teixeira, Helena Carla Castro

**Affiliations:** 1 Programa de Pós-Graduação em Ciências e Biotecnologia (PPBI), Instituto de Biologia, Universidade Federal Fluminense, Niterói 24210-130, RJ, Brazil; rebecaccpereira@gmail.com (R.C.C.P.); luterra.santos@gmail.com (L.T.); 2 Laboratório de Trombose e Câncer, Instituto de Bioquímica Médica Leopoldo de Meis, Universidade Federal do Rio de Janeiro, Rio de Janeiro 21944-970, RJ, Brazil; andrebiouff@gmail.com; 3 Programa de Pós-Graduação em Patologia, Hospital Universitário Antonio Pedro, Universidade Federal Fluminense, Niterói 24210-130, RJ, Brazil; 4 Laboratório de Modelagem Molecular e Pesquisa em Ciências Farmacêuticas—LAMCIFAR, NUPEM, Universidade Federal do Rio de Janeiro, Campus Macaé, Rio de Janeiro27965-045, RJ, Brazil; abreu_pa@yahoo.com.br

**Keywords:** marine products, molecular docking, diterpenes, *Dictyota menstrualis*, anticoagulant, antithrombotic

## Abstract

Thrombosis related diseases are among the main causes of death and incapacity in the world. Despite the existence of antithrombotic agents available for therapy, they still present adverse effects like hemorrhagic risks which justify the search for new options. Recently, pachydictyol A, isopachydictyol A, and dichotomanol, three diterpenes isolated from Brazilian marine brown alga *Dictyota menstrualis* were identified as potent antithrombotic molecules through inhibition of thrombin, a key enzyme of coagulation cascade and a platelet agonist. Due to the biotechnological potential of these marine metabolites, in this work we evaluated their binding mode to thrombin in silico and identified structural features related to the activity in order to characterize their molecular mechanism. According to our theoretical studies including structure-activity relationship and molecular docking analysis, the highest dipole moment, polar surface area, and lowest electronic density of dichotomanol are probably involved in its higher inhibition percentage towards thrombin catalytic activity compared to pachydictyol A and isopachydictyol A. Interestingly, the molecular docking studies also revealed a good shape complementarity of pachydictyol A and isopachydictyol A and interactions with important residues and regions (e.g., H57, S195, W215, G216, and loop-60), which probably justify their thrombin inhibitor effects demonstrated in vitro. Finally, this study explored the structural features and binding mode of these three diterpenes in thrombin which reinforced their potential to be further explored and may help in the design of new antithrombotic agents.

## 1. Introduction

In recent decades, several studies described the biotechnological potential of marine organisms metabolites in industry and medicine. These metabolites have been reported with different biological activities including antiplatelet, anticoagulant, and antithrombotic activities [1,2]. According to the World Health Organization, these biological profiles are of interest given that cardiovascular diseases, including thrombosis, are the main cause of death and incapacity in the world and the available treatments present several adverse effects [3].

**Figure 1:**
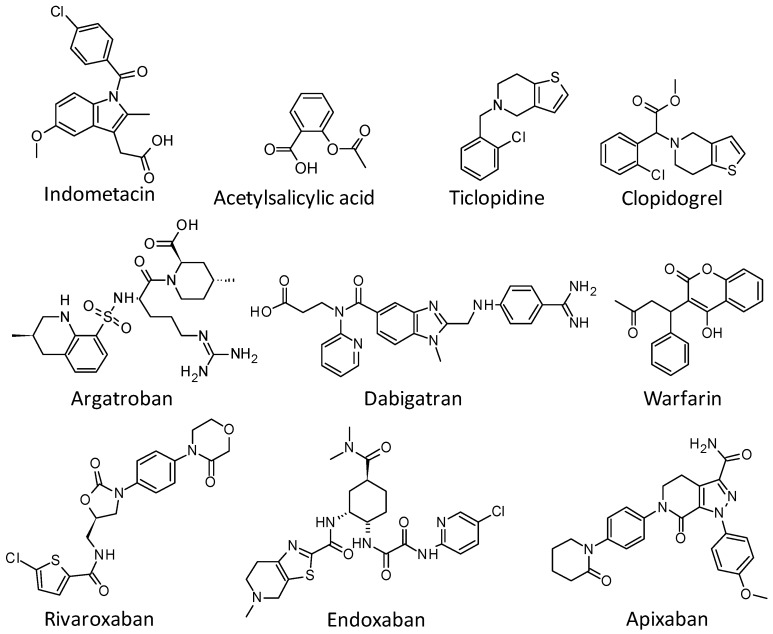
Antithrombotic agents used in clinics. Some studies described marine products from seaweeds as feasible anticoagulants to be explored such as those isolated from the brown alga, *Canistrocarpus cervicornis* (e.g., heterofucans) [9] and fruits of *Ilex paraguariensis* [10]. Diterpenes also represent a class of secondary metabolites with high biotechnological potential [11]. Recently, De Andrade Moura and coworkers reported the inhibitory effects against human platelet aggregation and blood coagulation of dichotomanol, a rare exclusively marine diterpene with two aldehyde groups and pachydictyol A and isopachydictyol A which are mainly prenylated derivatives of known guaiane sesquiterpenes, all isolated from the Brazilian marine brown alga *Dictyota menstrualis* [2] (Figure 2). As detected by in vitro enzymatic assays, these diterpenes act as anticoagulants and antiplatelets through interaction with Thrombin, a key enzyme of the coagulation cascade, a platelet aggregation agonist and an important target for thrombotic diseases treatment [2,12,13].

The current antithrombotic drugs therapies for cardiovascular diseases are based on different mechanisms including: (a) inhibition of arachidonate-induced platelet aggregation via blockage of cyclooxygenase 1 activity, (e.g., aspirin, indomethacin); (b) blocking ADP-binding receptor P2Y12 on platelet surface (e.g., thienopyridines such as clopidogrel, ticlopidine) [4]; (c) vitamin K antagonists (e.g., warfarin); (d) factor Xa inhibition (e.g., rivaroxaban, apixaban and edoxaban); and (e) Direct Thrombin Inhibition-DTI (e.g., argatroban and dabigatran) (Figure 1) [5]. These therapies still present limitations and side effects including bleeding and resistance development [4,6,7]. Therefore, the high mortality rates and the difficulties faced by these current therapies prompt the discovery of novel antithrombotic agents including those from marine environments [8].

Thrombin is an important serine protease from chymotrypsin family that presents a catalytic triad (His57, Asp102, and Ser195) in the active site, a substrate recognition loop (60-loop) with 8–9 insertion residues (Leu59-Asn62), and an autolysis-loop (γ-loop) formed by Leu144-Gly150 residues [14,15,16], all crucial for proper substrate binding and turn-over. Thrombin 60-loop bears hydrophobic residues that modulate the interactions with aromatic residues located at P3 position relative to the scissile bond, whereas γ-loop is more hydrophilic and flexible, and accommodates the C-terminal region of the substrate [17]. This enzyme is also regulated by a Na^+^ binding loop (Cys220-Tyr225) that positively modulates the enzymatic activity towards fibrinogen. Thus the procoagulant activity of thrombin is favored by Na^+^ binding, while in the absence of Na^+^, this enzyme goes through a shifts on selectivity towards protein C, whose activation leads to the degradation of factors VIIIa and Va with a final anticoagulant effect [16,17].

Thrombin presents two different anion binding exosites, including the fibrinogen/fibrin recognition site called anion-binding exosite I (ABE-I or exosite 1), and the heparin binding site also known as anion-binding exosite-II (ABE-II) or exosite 2. ABE-I surface contains positively-charged residues in the loops Phe34-Leu39 (34-loop) and Lys70-Glu80 (70-loop) and is the current target region of some antithrombotic drugs. ABE-II is also a known therapeutic target region, even more positively charged (Arg93, Lys236, Lys240, Arg101, and Arg233) despite its hydrophobic cleft [18,19].

Lately, the biotechnological development in the field of marine products have explored potential applications on thrombotic related pathological diseases [2,11,20,21]. In 2014 De Andrade Moura and co-workers described three diterpenes from *Dictyota menstrualis* (pachydictyol A, isopachydictyol A and dichotomanol) as inhibitors with direct effect on Thrombin catalytic activity against its natural (fibrinogen) and synthetic (chromogenic-S2238) substrates [2]. Therefore, these diterpenes are able to inhibit coagulation cascade and platelet aggregation, two important pathways targets for treating thrombotic related diseases. Although these diterpenes were described as thrombin inhibitor, there is no information about the molecular mechanisms of the ligand-receptor binding.

Thus, in this work we used molecular modeling approach in order to identify the key interactions and structural features responsible for the thrombin inhibitory effects of pachydictyol A, isopachydictyol A, and dichotomanol and to help in exploring the biotechnological potential of these diterpenes.

## 2. Results and Discussion

Thrombin has been an important target for the treatment of thrombosis and related diseases. Its inhibition by parenteral agents (e.g., heparin and low molecular weight heparins; recombinant hirudin, lepirudin and hirugen; synthetic Direct Thrombin Inhibitor—DTIas argatroban) and by others such as the oligopeptide bivalirudin have been important for treating several thrombosis-related diseases such as venous thromboembolism, and prevention on medical cardiovascular procedures (e.g., percutaneous coronary interventions) [13,22,23,24].

New findings about antithrombotic molecules are always in progress worldwide but most have been unsuccessful, such as ximelagatran (AstraZeneca) [25] that was withdrawn due to hepatotoxicity effects. Dabigratan is an oral DTI licensed for the prevention of thromboembolism and stroke worldwide [26,27] but it is still not known how long it will be effective, as side effects and resistance have been described for some anticoagulant/antiplatelet molecules after long periods of use (e.g., acetyl salicylic acid and clopidogrel) [28,29,30].

Factor Xa inhibitors have emerged as an interesting and promising therapy as anticoagulant. Targeting the point of convergence of intrinsic and extrinsic pathways enables the inhibition of thrombin production from both pathways [31,32]. Although they are an attractive alternative to warfarin, there are problems as in other therapies, in case of the non-vitamin K antagonist oral anticoagulants (including DTI and factor Xa inhibitors), difficulties may be faced when urgent reversal is needed as in case of bleeding or emergence procedure and the cost-effectiveness should also be considered [33,34].

In this context, the search for new antithrombotics is still an urgent need and marine products may represent a source for the screening of new agents.

### 2.1. Diterpenes Structure-Activity Relationship Analysis

**Figure 3:**
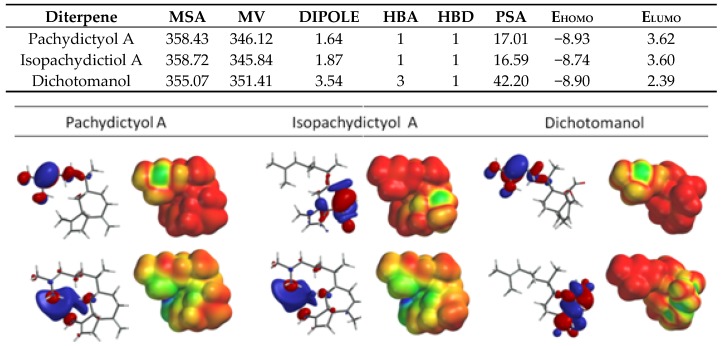
Stereoelectronic parameters of pachydictyol A, isopachydictyol A, and dichotomanol including Molecular Surface Area (MSA = Å^2^), Molecular Volume (MV = Å^3^), Dipole moment (debye), number of hydrogen bond acceptors/donors (HBA and HBD), Polar surface area (PSA = Å^2^), and HOMO and LUMO energies (E_HOMO_ and E_LUMO_ = eV) and HOMO (up) and LUMO (down) coefficient distribution and density maps. In the density map (right), the lowest orbital density areas are in red and the highest are in blue.

Due to the importance of water solubility and electrostatic complementarity for a feasible ligand-protein interaction, in this study we first evaluated the stereoelectronic and physicochemical properties of all three diterpenes to identify a correlation with their inhibitory profile. De Moura et al. (2014) tested the thrombin inhibitor effects of these three diterpenes showing better results for dichotomanol that caused 50% inhibition of the catalytic activity at 0.35 mM while pachydictyol A/isopachydictyol showed 50% inhibition at 0.68 mM [2]. Thus, we evaluated several parameters of these molecules including molecular surface area (MSA), molecular volume (MV), dipole moment, number of hydrogen bond acceptors (HBA) and donors (HBD), polar surface area (PSA), and HOMO and LUMO energies (E_HOMO_ and E_LUMO_) as well as HOMO and LUMO distribution maps (Figure 3).

The overall evaluation of the electronic properties of the diterpenes revealed that dichotomanol, the most active diterpene, has four-fold higher dipole moment in comparison to pachydictyol A and isopachydictyol A isomers (Figure 3). This feature is in agreement not only with dichotomanol higher polar surface area among the diterpenes evaluated, but also with DTIs chemical characteristics since mostly, they are molecules that interact with the acid/polar catalytic site of thrombin (Figure 3). We also analyzed the major microspecies in pH 7.4 but the theoretical study showed no ionized microspecies at physiological pH, suggesting that thrombin inhibition is performed by these molecules in a neutral state.

**Figure 4:**
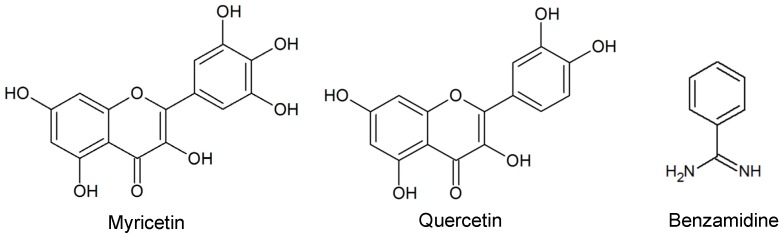
Serine proteases inhibitors described in literature.

The global analysis of HOMO and LUMO energies revealed that dichotomanol has the lowest HOMO and LUMO energies when compared to the prenylated guaiane isomers isopachydictyol A and pachydictyol A (Figure 3). This result indicated that this diterpene is more susceptible to a nucleophilic attack by electron-rich nucleophiles. Thrombin presents a highly negatively charged surface at the catalytic site, thus making it an electron-rich environment attractive to electrophilic molecules such as these diterpenes and flavonoids described on the literature (Figure 4) [35].

HOMO distribution was differently located at pachydictyol A, and isopachydictyol A changing from the region of exocyclic double bond in pachydictyol A, to endocylic bond in five-member ring in isopachydictyol A. On the other hand, the dichotomanol is a bicyclic diterpenes with two aldehydes, an α,β-unsaturated (Figure 3) which may lead to different binding modes, and complementary interaction to thrombin. In contrast, LUMO is kept concentrated at the same location at pachydictyol A and its isomer but not in dichotomanol (Figure 3). This specific dichotomanol HOMO and LUMO distribution probably favors its binding to thrombin, justifying the best inhibitor effect of dichotomanol detected by De Moura et al. (2014) using in vitro assays [2].

Many molecules from natural extracts (e.g., flavonoids) have been described with high susceptibility to nucleophilic attacks from proteases [36] such as flavonoids myricetin and quercetin also recently described in *Himanthalia elongate* (Figure 4) [37]. According to the literature, some of these flavonoids present a significant inhibitory profile against thrombin due to the presence of hydroxyl groups that mediate binding to thrombin aminoacid residues. Moreover, thrombin inhibition has also been shown to be positively correlated to the number of hydroxyl groups present in the flavonoid moiety [38]. This particular feature has been correlated with the inhibition of multi-subunit proteases complex such as the proteasome [36] and smaller serine proteases such as thrombin and Dengue virus NS2B-S3 [28,39].

**Figure 5:**
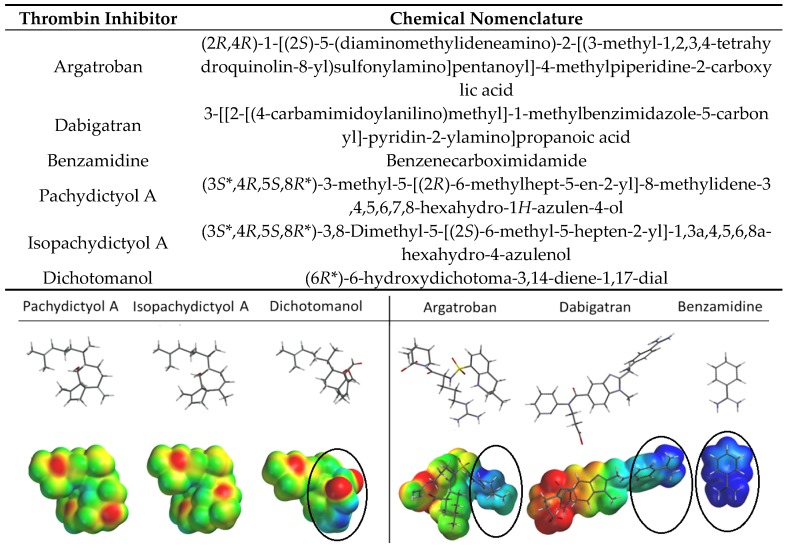
Comparison of chemical composition, 3D structure and electrostatic potential maps of pachydictyol A, isopachydictyol A, and dichotomanol with Direct Thrombin Inhibitors-DTI (argatobran and dabigratan), and the serine protease classic inhibitor benzamidine. The putative binding region of the most active diterpene dichotomanol and the binding region of DTIs and benzamidine are circled in black. The electrostatic potential maps range is from −80 to 140 kJ/mol.

As expected, the comparison of these diterpenes with other DTIs, such as argatroban and dabigratan, as well as with the classic serine protease inhibitor benzamidine revealed different electrostatic potential distribution due to their different chemical group composition (Figure 4 and Figure 5). However, the most active diterpene, dichotomanol, showed a positive region similar to these thrombin inhibitors, which is due to the presence of α,β-unsaturated dialdehydes (Figure 5) probably involved on binding to thrombin. Overall, these data are not only in agreement to the in vitro assays reported by De Moura and coworkers but also reinforced the flexibility of thrombin that is able to interact with significant selectivity with different ligands from the human hemostatic system as well as with chemically different inhibitors, including marine products.

### 2.2. Diterpenes Molecular Docking Evaluation

**Figure 6:**
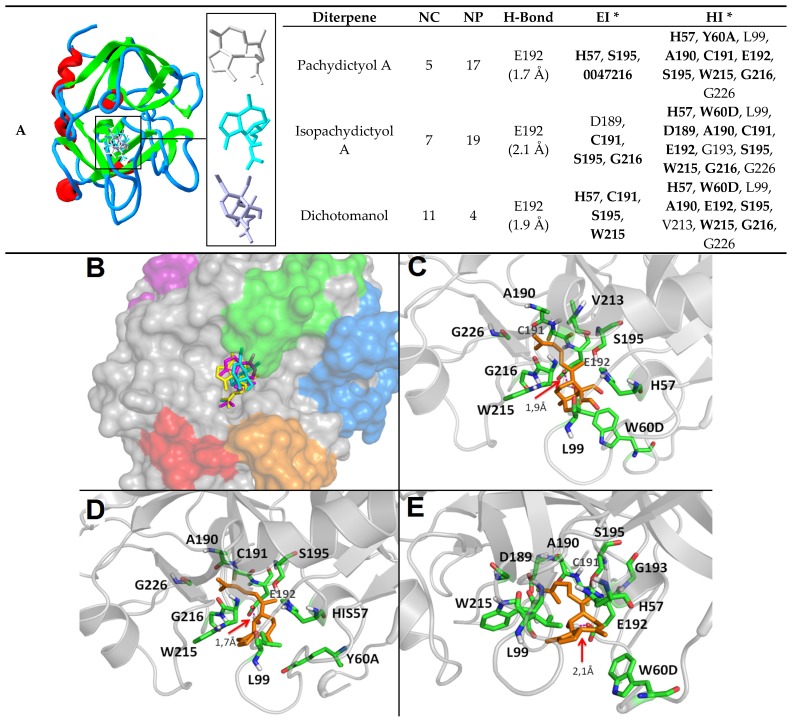
Comparison of 3D structure and docking data complexes of pachydictyol A, isopachydictyol A, and dichotomanol with human alpha-thrombin catalytic site (PDB: 1PPB). (**A**) The 3D-structural alignment reveals the diterpenes localization within the catalytic site and the different conformation of each diterpene (pachydictyol A, light gray; isopachydictyol A, cyan; dichotomanol, purple) (right) as well as the parameters analyzed including number of clusters (NC) and poses (NP) on the lowest energy cluster, hydrogen-bond (H-bond), electrostatics interactions (EI) and hydrophobic interactions (HI) (left). (**B**) Diterpene proximity to thrombin binding regions including ABE-I (marine), ABE-II (purple), Na+ binding site (red), 60-loop (green), and γ-loop (orange). Zoom of the diterpenes (orange) dichotomanol (**C**); pachydictyol (**D**); isopachydictyol (**E**) bound to thrombin (green) with arrow pointing to the most important hydrogen bond (magenta). * **Bold** = thrombin residues that also interact with the flavonoids myricetin and quercetin according to Mozzicafreddo et al. 2006 [28].

Shape complementarity is an important parameter for ligand-protein recognition that rules many successful drug-development strategies such as fragment-based drug design [40]. According to our docking analyses of the diterpenes in thrombin catalytic site, dichotomanol has improved water solubility and increased electrostatic complementarity to thrombin active site in comparison to pachydictiol a and isopachydictiol A (Figure 6). Based on a recent data from successful thrombin inhibitors, highly watersoluble molecules with low electronic density such as argatroban and dabigatran are examples of successful small active-site inhibitors for thrombin, analogous to these diterpenes [24].

**Figure 7:**
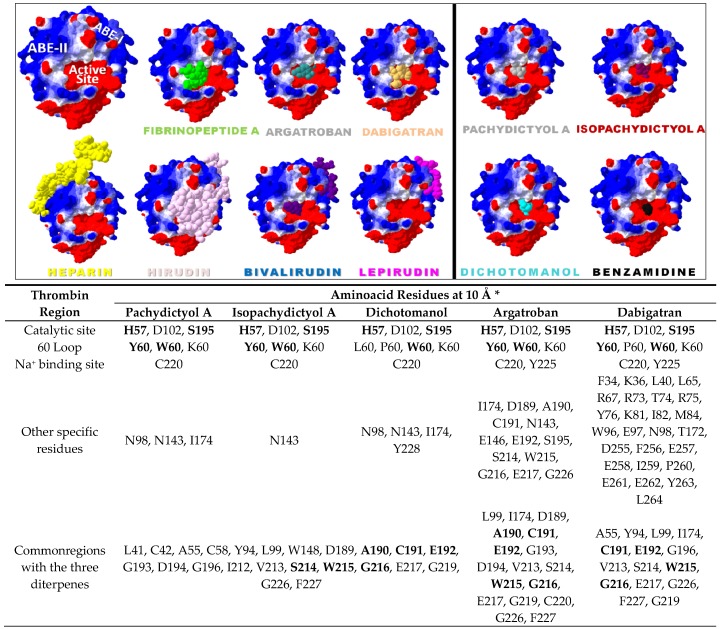
Comparison of 3D representation and residues interaction network of the complexes involving diterpenes or known anticoagulants with thrombin. The electrostatic potential map (blue = positively charged, red = negatively charged) shows thrombin catalytic site and the binding regions: anion binding Exosite I also known as Exosite 1 or fibrin binding site (ABE-I) and anion binding Exosite II also known as Exosite 2 or Heparin Binding site (ABE-II) and the ligand localization including fibrinopeptide A (from natural substrate Fibrinogen), argatroban and dabigatran (small inhibitors known as DTI), heparin (indirect inhibitor naturally produced by mast cells and basophiles that binds to ABE-II), hirudin (a peptide from saliva of leech *Hirudo medicinalis* that binds to catalytic site and ABE-I), bivalirudin (a synthetic molecule based on hirudin with four glycines connecting two amino acids sequences that acts as a divalent DTI), and lepirudin (a mutant hirudin-N-terminal LEU/ILE-without TYR63 sulfate group which binds only to ABE-I). All three diterpenes bind directly to thrombin catalytic site similar to the univalent DTIs (argatroban and dabigatran) and the classical serine protease benzamidine (up). The enzyme residues are in the interaction network at 10 Å of the ligand and they are in accord with trypsin numbering. * **Bold** = thrombin residues that also interact with the flavonoids myricetin and quercetin according to Mozzicafreddo et al. 2006 [28] (down).

The analysis of the overall binding energy, number of clusters, and number of poses from the most stable cluster showed all diterpenes interacting with thrombin catalytic site. Interestingly, the binding complex of dichotomanol with thrombin catalytic site (Binding energy, BE = −3.31 kcal/mol) showed the dichotomane ring at the S2 pocket (Figure 6). Tight hydrogen bonds were detected between the (2)-hydroxyl group of this diterpene with Glu192 (1.9 Å) residue (Figure 6 and Figure 7). This stable pose was also mediated by electrostatic interactions with His57, Cys191, Trp215, and particularly the nucleophile Ser195, that lies within 4 Å from the electrophilic dichotomane ring, in a distance suitable for nucleophilic attack (Figure 6 and Figure 7). These data indicated the formation of high-stable complexes as suggested for many flavonoids with the proteasome [36]. Hydrophobic interactions with His57, Trp60D, Leu99, Ala190, Glu192, Ser195, Val213, Trp215, Gly216, and Gly226 residues also recruited residues from the S3 pocket including residues from the 60-loop (Figure 6 and Figure 7), which is known as important for thrombin recognition by small synthetic inhibitors [41].

Although differences were observed among the stereoelectronic properties of pachydictyol A and isopachydictyol A in the structure-activity relationship analysis, the molecular docking evaluation revealed the formation of an improved ligand-protein complex between isopachydictyol A and thrombin in comparison to pachydictyol A. This result suggested that the different HOMO distribution of isopachydictyol A (Figure 3) may provide a structure of higher shape complementarity to a thrombin active site, prompting to a further analysis of this particular tautomeric form, including the planning of new prototypes and drug-design strategies based on this molecule. Isopachydictyol A-thrombin complex (BE = −5.2 kcal/mol) showed a hydrogen bond with Glu192 (2.1Å) and electrostatic interactions with Asp189, Ala190, Cys191, Glu192, Gly193, Ser195, Trp215, Gly216, and Gly226 that also included Ser195 at a distance similar to that observed with dichotomanol. In addition, hydrophobic interactions with His57, Leu99, Cys191, Gly193, Ser195, Val213, Trp215, and Gly216 reinforced the binding of this molecule to the thrombin region S2 (Figure 7).

Some of the thrombin residues involved in isopachydictyol A-thrombin complex were also observed when the tautomer pachydictyol A was bound to this enzyme (BE = −5.05 kcal/mol). Several electrostatic interactions with His57, Ser195, and Gly216, as well as hydrophobic interactions with His57, Tyr60A, Leu99, Ala190, Cys191, Glu192, Trp215, Gly216, and Gly226 residues and a hydrogen bond with Glu192 (1.7 Å) residue were also detected reinforcing once more the in vitro assays (Figure 6 and Figure 7).

Overall, docking analysis suggested that all diterpenes stay within the S2 pocket of the enzyme, near from theSer195. This competition may avoid the substrate binding at a feasible distance for a nucleophilic attack by OH group of the Ser195 (Figure 6). The highest dipole moment, polar surface area and lowest electronic density of dichotomanol may improve hydration and aqueous availability while favoring electrostatic complementarity to the thrombin active site, in comparison to pachydictyol A and isopachydictyol A (Figure 6). This feature may lead to the higher inhibition rate observed in vitro for dichotomanol towards thrombin. However, a better shape complementarity is shown for both isopachydictyol A and its tautomeric isomer, pachydictyol A, which theoretically explain their active profile. We also searched for an interaction network involving thrombin loops and regions important for the enzyme catalytic activity at a distance of 10 Å of the marine ligands (Figure 6 and Figure 7). Our results showed aminoacid residues from the thrombin 60-loop as well as the main catalytic residues His57, Asp102, and Ser195 interacting directly with them (Figure 6 and Figure 7) but none from the γ-loop or Na^+^ binding site (not shown).

Despite the in vitro identification of these diterpenes as univalent DTI, which pointed to the catalytic site as the unique binding site for these molecules, we also briefly explored the feasibility of these molecules on binding to ABE-I or II. Our comparison of binding energy level of these three diterpenes docking complexes with thrombin binding regions (Catalytic site, ABE-I, or ABE-II) pointed once more the catalytic site as the most feasible target region for each diterpene in agreement to the in vitro data due to the lowest binding energy level detected among them (data not shown). The methodology was validated by redocking the inhibitor D-Phe-Pro-Arg chloromethylketone into the active site of human alpha-thrombin (PDB: 1PPB) (RMSD = 1.11 Å) with the ligand superimposed to the native co-crystallized enzyme with a free energy (∆G) of −10.46 kcal/mol (not shown).

Interestingly, the comparison of our molecular dockings complexes with other anticoagulants that target thrombin such as heparin, hirudin, argatobran, and dabigratan reinforced pachydictyol A, isopachydictyol A, and dichotomanol as univalent DTIs (Figure 7) as previously indicated [2]. Comparison of thrombin aminoacid residues involved in the diterpene interaction network with the DTIs argatobran and dabigratan revealed that diterpenes interact with many residues that are involved in argatroban inhibitory mechanism (e.g., His57, 60-loop residues, Asp189, Ser195, and Trp215 residues) (Figure 6 and Figure 7) [42]. Dabigatran presents a different inhibition mechanism, but it also shows some similarity, which reinforced several interaction possibilities when it comes to inhibiting thrombin [43]. The similar but not identical mechanism of our diterpenes pointed to these marine molecules as potential antithrombotic prototypes that present important structural features that should be further explored for thrombin inhibitor design.

Currently, most of the antithrombotic profile described for natural products such as marine metabolites, particularly diterpenes, are not structurally analyzed [2,44]. However, the literature recently described the flavonoids myricetin and quercetin bound to thrombin catalytic site [38]. The comparison of the molecular dockings of our diterpene complexes with these flavonoids complexes report revealed differences including interactions with S1, S2, and S3 pockets (Figure 6 and Figure 7). Myricetin interacted with Ala190, Cys191, Glu192, Gly216, Lys60, Ser195, Ser214, Trp215, and Trp60 residues, whereas quercetin interacted with Ala190, His57, Lys60, Gly216, Ser214, and Trp60 residues (trypsin numbering) [38]. Interestingly, our diterpenes apparently presented more interactions and residues involved in the maintenance of the complex with thrombin than these flavonoids, which also might be explored in planning new antithrombotic molecules (Figure 6 and Figure 7).

## 3. Materials and Methods

### 3.1. Structure-Activity Relationship Analysis

The molecules were submitted to the default systematic conformational analysis procedure, available in the SPARTAN’10 software package (Wavefunction Inc., Irvine, CA, USA, 2000), using molecular mechanics and the MMFF force field and the most stable conformers were used for further calculations.

In order to evaluate the stereoelectronic properties, all structures were submitted to a full geometry optimization process using the Recife Model 1 (RM1) semi-empirical method, and finally the stereoelectronic properties were calculated with Hartree-Fock method using the basis set 6-311G* available in Spartan’10. Then, we calculated the electronic properties including Highest Occupied Molecular Orbital (HOMO) and Lowest Unoccupied Molecular Orbital (LUMO) energy, density maps, orbital coefficients distribution, molecular dipole moment and molecular electrostatic potential maps (MEP) of each compound.

Further, major ionized microspecies, water solubility, and number of hydrogen bond acceptors and donors were carried out with Chem Axon Calculator server (Copyright © 1998–2017 ChemAxon Ltd., Budapest, Hungary). All these structural features and calculated properties were used to correlate with the thrombin catalytic activity of these three diterpenes described in the literature by Moura et al., 2014 [2].

### 3.2. Molecular Docking Study

The molecular docking study was carried out with AutoDock 4.2 with AutoDockTools 1.5.6 extension. Dockings were performed with the ligands dichotomanol, pachydictyol A, and isopachydictyol constructed in the Spartan program and the human Alpha thrombin, available on PDB with the entry code 1PPB. The protein and the ligands were prepared by the addition of hydrogens atoms, Gasteiger partial atomic charge calculation, polar hydrogen graphic analyses, and Ad4 program file atoms definition. The maximum allowed conformation number was defined in the active site.

The cubic grid box was set to 60 × 60 × 60 points with a spacing of 0.375 Å containing the main residues of catalytic site, loop-60 and γ-loop regions, Na^+^ binding site, and ABEI or ABEII. In case of the catalytic site the grid box were centralized using the following coordinates (*x* = 3.178; *y* = 21.109; *z* = 15.649). To find the best orientations and conformations of the ligands in the protein binding sites the Lamarckian Genetic algorithm was selected, with an initial population size of 150, a maximum number of evaluations of 2.5 × 10^6^ (medium), maximum number of generations of 27,000, gene mutation rate of 0.02, crossover rate of 0.8, and number of GA runs equal to 50.

In order to confirm the efficiency of this methodology, docking performance and accuracy were validated by re-docking the inhibitor D-Phe-Pro-Arg Chloromethylketone in the human alpha-thrombin active site (PDB:1PPB).

## 4. Conclusions

Marine organisms represent a valuable source of molecules, particularly diterpenes, with high biotechnological potential (as in antithrombotic activity, for example) still to be further explored. In this work, we evaluated three diterpenes (dichotomanol, pachydictyol A, and isopachydictyol A) isolated from the Brazilian marine brown alga *Dictyota menstrualis* in silico with thrombin inhibitor effects already described. Structure-activity relationship and molecular docking evaluations performed with human alpha-thrombin showed the unique chemical features of these diterpenes and pointed the catalytic site of this enzyme as the potential binding site. The in silico studies also justified the potential of the most active diterpene, dichotomanol, due to its higher polar surface area and dipole moment, as well as HOMO and LUMO specific distribution that account for a better water solubility and favorable electrostatic interactions with thrombin active sites. Enhanced complementarity to thrombin catalytic sites is also achieved by pachydictyol A and isopachydictyol A. Although these studies do not guarantee that any of these diterpenes will be a new option for treatment or prophylaxis of thrombosis or clotting disorders in the future, our data may open new possibilities. The knowledge obtained here about the structure-activity of these diterpenes and their binding sites and interactions with thrombin may facilitate ligand-based drug design (LBDD) and structure-based drug design (SBDD) and the use of medicinal chemistry strategies for molecular modifications (e.g., bioisosterism, simplification, hybridization) especially in dichotomanol. Overall, these data may add to future in vitro and in vivo studies for the development of new and more active antithrombotic prototypes based on these marine metabolites.

## Figures and Tables

**Figure 2:**
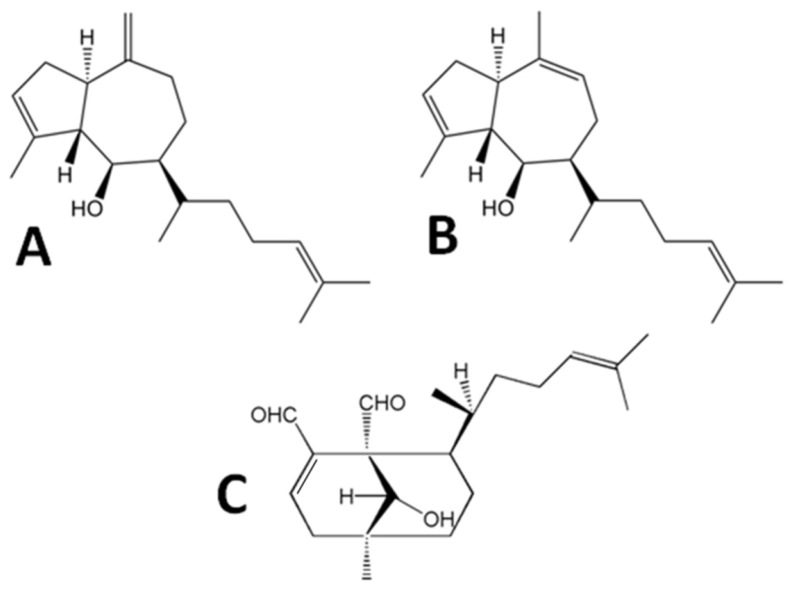
Diterpenes isolated from Brazilian marine brown alga *Dictyota menstrualis*. (**A**) pachydictyol A; (**B**) isopachydictyol A; and (**C**) dichotomanol.
